# Iron metabolism in a mouse model of hepatocellular carcinoma

**DOI:** 10.1038/s41598-025-86486-x

**Published:** 2025-01-16

**Authors:** Dilay Yilmaz, Umesh Tharehalli, Rossana Paganoni, Paul Knoop, Andreas Gruber, Yuexin Chen, Rui Dong, Frank Leithäuser, Thomas Seufferlein, Kerstin Leopold, André Lechel, Maja Vujić Spasić

**Affiliations:** 1 Institute of Comparative Molecular Endocrinology, Ulm University, 89081 Ulm, Germany; 2 Department of Internal Medicine I, University Hospital Ulm, Albert-Einstein-Allee 23, 89081 Ulm, Germany; 3 Institute of Analytical and Bioanalytical Chemistry, Ulm University, Ulm, Germany; 4 Department of Pathology, University Hospital Ulm, Ulm, Germany

**Keywords:** Iron metabolism, Trace elements, Liver cancer, Hepcidin, Transferrin receptor, p53, Cancer models, Mechanisms of disease, Homeostasis

## Abstract

Hepatocellular carcinoma (HCC) remains the most prevalent type of primary liver cancer worldwide. p53 is one of the most frequently mutated tumor-suppressor genes in HCC and its deficiency in hepatocytes triggers tumor formation in mice. To investigate iron metabolism during liver carcinogenesis, we employed a model of chronic carbon tetrachloride injections in liver-specific p53-deficient mice to induce liver fibrosis, cirrhosis and subsequent carcinogenesis. A transcriptome analysis of liver carcinoma was employed to identify p53-dependent gene expression signatures with subsequent in-depth analysis of iron metabolic parameters being conducted locally within liver cancers and at systemic levels. We show that all mutant mice developed liver cancer by 36-weeks of age in contrast to 3.4% tumors identified in control mice. All liver cancers with a p53-deficient background exhibited a local iron-poor phenotype with a “high transferrin receptor 1 (*Tfr1*) and low hepcidin (*Hamp*)” signature. At systemic levels, iron deficiency was restricted to female mice. Additionally, liver tumorigenesis correlated with selective deficits of selenium, zinc and manganese. Our data show that iron deficiency is a prevalent phenomenon in p53-deficient liver cancers, which is associated with alterations in *Hamp* and *Tfr1* and a poor prognosis in mice and patients.

## Introduction

Liver cancer is the sixth most frequently diagnosed type of cancer and the third leading cause of cancer-related mortality worldwide^1^. The most common type of primary liver cancer is the hepatocellular carcinoma (HCC), which accounts for 75–85% of all liver cancers and more than 800,000 deaths/year worldwide^1–3^.

Findings from studies on whole-genome and the exome sequencing of 243 human liver tumors identified the *TP53* (*Trp53* in mice; short p53*)* as the most commonly mutated tumor suppressor gene in HCC samples and the p53 pathway being affected in half of HCC patients^4–6^. The importance of p53 as a tumor suppressor is evident by its ability to act as a transcription factor to regulate the expression of a series of target genes necessary for cell survival and death^7^. Mutated p53 is accumulated in the nuclei of transformed cells and is associated with a poor prognosis in HCC^8,9^. Preclinical animal models revealed that liver-specific deletion of p53 in mice led to tumor formation, demonstrating that p53 deficiency, as a single genetic lesion, induced liver tumors with high penetrance^10^. Interestingly, p53 deletion was not sufficient to induce tumors in the brain and intestine, indicating organ-specific differences concerning the role of p53 in tumor development^11,12^.

Emerging evidence indicates that deregulation of p53 leads to several metabolic disorders that are pivotal during cancer progression (reviewed in^13^). Importantly, p53 was shown to regulate several key components of iron metabolism^14–18^ and, vice versa, p53 activities were modulated by intracellular iron levels and heme^14,15,17,19^. For example, studies performed in human hepatoma HepG2 cell line showed that p53 activation correlated with the induction of iron hormone hepcidin, and that p53 controlled hepcidin transcription by binding to putative p53-responsive elements in the promoter of the hepcidin (*Hamp*) gene (at position -435/-413 bp)^17^. Hepcidin can thus be activated by p53 and abated when p53 is silenced in vitro^17^. So far, no studies have yet corroborated p53-mediated hepcidin regulation in vivo*.* Considerably less is known regarding the mechanisms by which p53 coordinates iron metabolism during liver cancer development.

Iron is an essential micronutrient and its levels in cells and organisms must be tightly regulated to prevent iron overload and iron deficiency. Cancer cells have high demand for iron that is required to sustain their high proliferative capacity, survival, tumor development and metastasis^20–22^. Increase in intracellular iron can promote tumor cell growth and proliferation by altering the expression of iron metabolism-related genes and proteins^21^. For example, exposing cancer stem cells to iron resulted in iron accumulation in the cells and subsequent transformation of cells into a more aggressive phenotype that was prone to metastasis; by contrast, starving cancer cells, with the use of iron-sequestering drugs, inhibited tumor growth^23,24^. This ‘iron addiction’ phenomenon of cancer cells is reflected in the higher transferrin-receptor 1 (TFR1) expression and enhanced iron uptake^25,26^ leading to increased levels of intracellular, metabolically active iron pool^20,21^. In addition, downregulation of iron-hormone hepcidin was observed in the early stages of HCC^27^ and low hepcidin levels correlated with tumor stage and cancer progression^28^. In particularly, increased expression of *TFR1* and decreased *HAMP* have been found in HCC in animal studies and more recently, in clinical samples^29,30^. Iron deficiency in liver tumors and the presence of iron-free preneoplastic lesions were observed in the conditions of hepatic siderosis in mice, rats and in patients with HFE-hemochromatosis^29–34^. This data shows that both, iron levels and the regulation of iron-metabolic molecules, are altered during carcinogenesis.

Given that p53 regulates several key components of iron metabolism^14–17^ and that p53 deficiency in mice induces tumor formation in the liver^10^, we sought to investigate iron metabolism in p53-deficient mice using a model of CCl_4_-induced liver carcinogenesis, which closely reproduces the steps of liver carcinogenesis in patients. The results presented here extend findings from previous studies showing that rapidly growing p53-deficient HCC tumors are at the status of iron deficiency and present low hepcidin and high TFR1 signatures. Moreover, liver carcinogenesis in female mutant mice associates with reduced circulating hepcidin levels and systemic iron deficiency.

## Results

### Establishing a mouse model of chronic liver damage and carcinogenesis

**Fig. 1:**
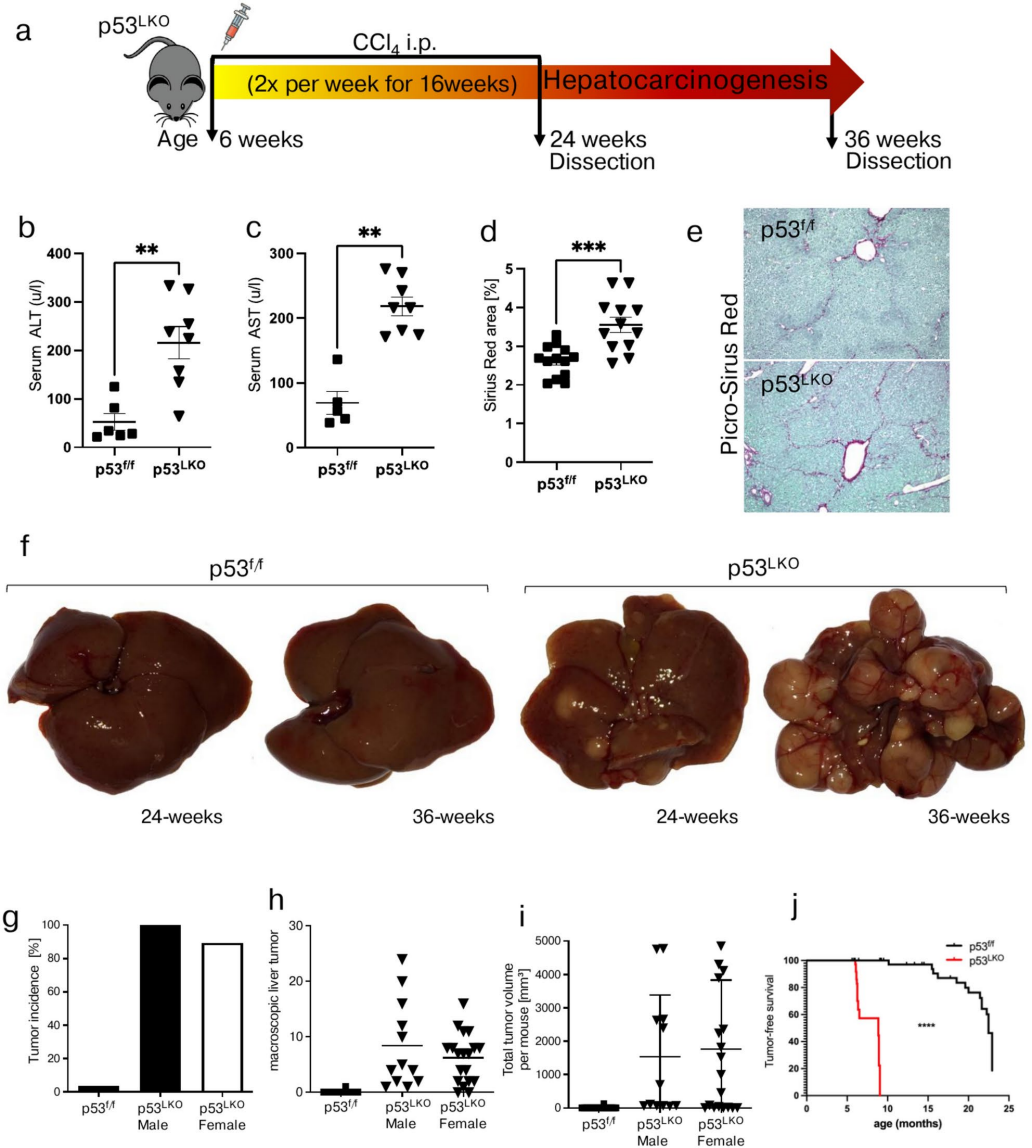
Liver carcinoma formation in p53^LKO^ mice is highly accelerated following chronic CCl_4_ treatment. (**a**) Study design. Mice with liver-specific Trp53 deletion (short: p53^LKO^) and p53^f^^/f^ control mice, both males and females, were injected intraperitoneally (i.p.) with carbon tetrachloride (CCl_4_; 0.5 ml/kg body weight) at 6 weeks of age, twice per week for total 16 weeks. Mice were sacrificed at 24 and 36 weeks of age. (**b, c**) Serum ALT (alanine transaminase) and AST (aspartate transaminase), indicative of liver damage in CCl_4_ treated mice (n = 6;8). (**d**) Bar graphs depict liver fibrosis analysed by Desmet stage (n = 17;11). (**e**) Representative photographs of Picro-Sirius-red staining of liver from p53^f^^/f^ mice (top) and of liver from p53^LKO^ mice (bottom). (**f**) Representative photographs of macroscopic liver carcinoma formation from p53^f^^/f^ and p53^LKO^ at the age of 24 and 36 weeks. (**g**) Bar chart represents tumor incidence in p53f^/f^ (n = 1/29) and p53^LKO^ male (n = 12/12) and female mice (n = 17/19). (**h, i**) Dot plots depict macroscopic tumor formation and total tumor volume per mouse in p53^f^^/f^ and p53^LKO^ male and female mice. (**j**) Tumor-free survival curve of p53^f^^/f^ and p53^LKO^ mice treated with chronic CCl_4_ injections.

The objective of this study was to investigate whether liver carcinogenesis is associated with impairments in iron metabolism. To this end, a mouse model was first established that mimics chronic liver damage in humans. Given that p53 is one of the most commonly mutated genes in human liver cancers^6^, we used mice with liver-specific knockout of p53 (further abbreviated as p53^LKO^)^10^ to induce chronic liver damage by long-term carbon tetrachloride (CCl_4_) treatment (Fig. 1a). We show that p53^LKO^ mice exhibited signs of chronic liver damage, as evidenced by elevated alanine aminotransferase (ALT) and aspartate aminotransferase (AST) and increased liver fibrosis compared to p53^f.^^/f^ mice (Fig. 1b-e, Suppl. Figure 1). Moreover, all p53^LKO^ male and 89.5% of female mice developed liver carcinoma by the age of 36 weeks (Fig. 1f, g). The tumor entity was confirmed by H&E and by immunohistochemical staining for HepPar1, as a marker for hepatocellular carcinoma, cytokeratin 7 (CK7), used as a marker for cholangiocarcinoma, and Picro-Sirius Red, as collagen stain (Supplementary Fig. 1). HCC development was seen in p53^f^^/f^ and in p53^LKO^ mice (Supplementary Fig. 1). The increase in tumor burden in p53^LKO^ mice was confirmed by an increased number of macroscopic liver carcinoma (7.06 tumor/p53^LKO^ mouse vs 0.03 tumor/p53^f^^/f^ mouse) and total tumor volume per mouse (Fig. 1h, i). In contrast to p53^LKO^ mice, tumor formation was significantly delayed in p53^/f^ control mice (Fig. 1j): up to the age of 90 weeks, the tumor incidence was 34.6% in males and 31.8% in female p53^f^^/f^ mice.

These data are in agreement with previous studies on carcinogen-induced liver cancer, which have shown that tumor formation is more prevalent in male mice than in female mice^35^, and that it can take up to two years for HCC to arise in wild-type background^36^.

### Comparative transcriptome analysis reveals altered iron metabolism in liver cancers with loss of p53

**Fig. 2:**
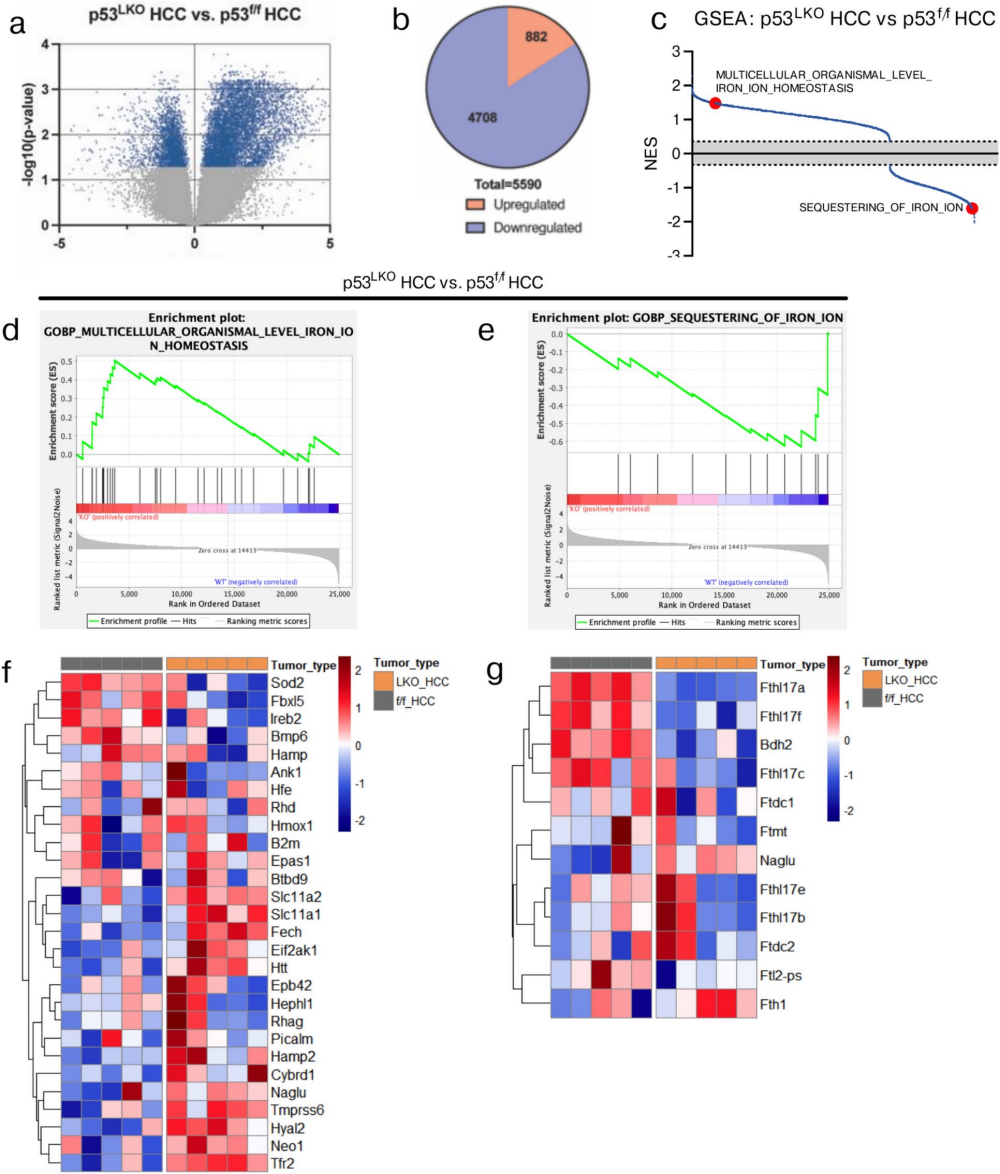
Transcriptome analysis of liver carcinoma reveals p53-dependent gene expression signatures associated with iron metabolism. Transcriptome analysis of liver carcinoma from p53^LKO^ and p53f.^/f^ male mice. (**a**) Volcano plot representation of genes that differ significantly in HCC in p53^LKO^ in comparison to HCC from p53f.^/f^ mice (n = 5;5). Plotted is significance (adjusted p-value) versus log2 fold change (FC) on the y and x-axis, respectively. Depicted in blue are genes significantly different with adjusted p < 0.05 and the FC > 2. (**b**) Pie charts illustrate the spectrum of upregulated and downregulated genes and the number of differentially regulated genes between HCC of p53^LKO^ and p53f.^/f^ mice. (**c**) GSEA pathways associated with iron metabolism are plotted in red. (**d, e**) Pre-ranked GSEA enrichment plots of representative signaling pathways with corresponding heat maps displaying genes grouped according to the expression intensities. Euclidean distance was used for hierarchical clustering. NES, normalized enrichment score; GSEA, gene set enrichment analysis. (**f, g**) Heat map of genes differentially regulated between HCC of p53^LKO^ and p53f.^/f^ mice in respective signaling pathways (**d, e**).

So far, we show that p53^LKO^ mice display a more aggressive tumor phenotype, reflected in accelerated tumor growth and development upon chronic CCl_4_ treatment, when compared to p53^f^^/f^ mice. Furthermore, the loss of p53 is associated with poor differentiation and reduced survival [37, Katz, 2012 #235]. Given these findings, we next investigated whether liver cancer development and progression, which were greatly enhanced by p53 deficiency as all mutant mice developed tumors, might be accompanied and potentially aggravated by changes in iron metabolism. To address this question, we employed two approaches. First, we performed a whole genome transcriptome comparison between HCC entities from p53^LKO^ and p53^f^^/f^ mice and have identified 5590 differentially expressed genes, with 882 upregulated genes and 4708 downregulated genes (with fold change > 2; p < 0.05) in p53^LKO^ HCC (Fig. 2a, b). The gene set enrichment analysis (GSEA) revealed the enrichment of several pathways associated with iron metabolism (Fig. 2c); the two most prominent are shown in Fig. 2d and e. Within these pathways, we identified several gene transcripts being upregulated in HCC from p53^LKO^ mice, including transferrin receptor, known for mediating the transport of di-ferric transferrin iron, and divalent metal iron transporters, *Slc11a1* and *Slc11a2* (*Nramp1* and *Nramp2*, respectively), and *Cybrd1* (*Dcytb1*) (Fig. 2f), while the expression of hepcidin and its upstream regulators (such as *Hfe* and *Bmp6*) was significantly downregulated (Fig. 2f). Several transcripts encoding for the ferritin heavy polypeptide-like members (*Fthl*), involved in iron binding activity and intracellular sequestering of iron, were downregulated in HCC from p53^LKO^ mice in comparison to HCC from p53^f^^/f^ mice (Fig. 2g). These results indicate that p53 loss in HCC alters the expression of major iron metabolic genes, which is potentially associated with increased iron uptake and utilization by p53-deficient cancer cells, required to sustain their high proliferative capacity and development into an aggressive phenotype.

### Liver carcinogenesis in p53 mutant mice produces iron deficient tumors characterized by ‘high TfR1 and low hepcidin’ signatures in comparison to adjacent non-tumor liver tissues

**Fig. 3:**
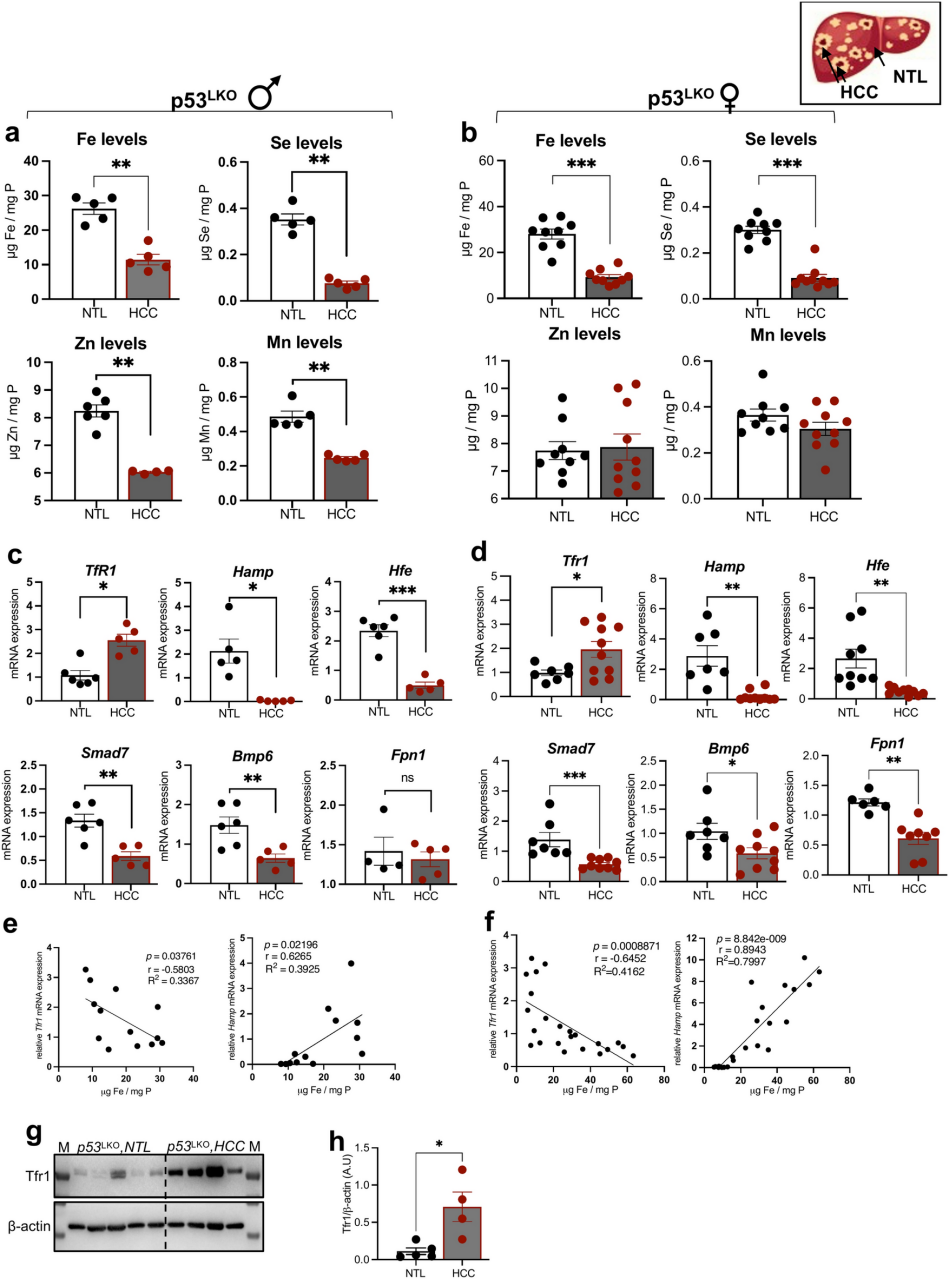
Liver carcinoma show a reduced trace element content and dysregulated iron metabolism. (**a, b**) Total trace element content (iron, selenium, zinc and manganese) in NTL and HCC of p53^LKO^ male and female mice, respectively. (**c, d**) mRNA expression levels of *Tfr1*, *Hamp* (hepcidin), *Hfe*, *Smad7*, *Bmp6*, and *Fpn1* in NTL and HCC of p53^LKO^ male and female mice, respectively. (**e, f**) Correlation analysis between *Tfr1, Hamp* and iron levels in HCC tissues from p53^LKO^ male and female mice, respectively. (**g, h**) Representative immunoblot analysis and quantification blots of TFR1 relative to β-actin levels (expressed in arbitrary units) in NTL and HCC of p53^LKO^ female mice.

In the second set of experiments, we conducted comprehensive systemic investigations *i)* to measure iron content in HCC and adjacent non-tumor liver tissues and, *ii)* to ascertain whether any discrepancies in iron metabolism may contribute to accelerated hepatocarcinogenesis observed in p53^LKO^ mice. To this end, we isolated hepatocellular carcinoma (HCC) and non-tumor liver tissues (NTL) from p53^LKO^ and p53f.^/f^ mice and first compared iron status among these two entities. Total iron levels in HCC and NTL tissues were measured using a highly sensitive total-reflection X-ray spectrometry (TXRF). We show that iron levels were on average 3-times lower in HCC tissues from p53^LKO^ male and female mice when compared to iron levels in NTL (Fig. 3a, b), demonstrating that CCl_4_-induced liver carcinogenesis in mice produces HCC with an iron-deficient status. In p53f.^/f^ mice, a tendency towards decreased iron levels in HCC was observed when compared to NTL, however, the data did not reach statistical significance because only two HCC-p53f.^/f^ samples were available for the analysis (Suppl. Figure 2a). In addition to iron, reduced levels of selenium (Se), zinc (Zn), and manganese (Mn) were measured in HCC from male p53^LKO^ and p53f.^/f^ mice in comparison to respective NTL tissues (Fig. 3a, Suppl. Figure 2b-d) . Similarly, CCl_4_-induced liver carcinogenesis in female p53^LKO^ mice produced HCC with low Fe and Se, while levels of Zn and Mn were not affected (Fig. 3b). This suggests that the metabolism of trace elements, including iron, selenium, zinc and manganese, may be important factor in hepatocarcinogenesis and may play a role in tumor proliferation. Consequently, the metabolism of trace elements may represent promising avenues for the treatment of liver cancer.

Further studies were focused to evaluate differences in iron metabolic regulators by comparing HCC and NTL tissues. Given that lack of p53 in HCC affects several pathways associated with iron metabolism (Fig. 2) and that liver tumors develop an iron-deficient phenotype, we postulated that iron uptake and utilization may be compromised. The analysis of *Tfr1* levels revealed a significant increase in HCC from p53^LKO^ mice when compared to the levels present in adjacent non-tumorous liver (NTL) and a reduction in mRNA expression of iron-hormone hepcidin, reaching almost undetectable levels in HCC tissues (Fig. 3c). In line with hepcidin, a decrease in mRNA expression levels of several key iron genes, including *Hfe*, *Smad7*, *Bmp6* was measured, while mRNA expression of ferroportin (Fpn1) was not significantly changed (Fig. 3c). Similar findings were obtained in HCC tissues from female p53^LKO^ mice: high *TfR1* and low mRNA expression of *Hamp*, *Hfe, Smad7, Bmp6*, and Ferroprotin (*Fpn1*), were observed in liver cancer tissues in comparison to NTL (Fig. 3d). Importantly, elevated *Tfr1* expression and low *Hamp* levels associated with an iron-poor phenotype of p53-deficient liver cancer when compared to NTC tissues (Fig. 3e-f), while these effects were significantly less pronounced in p53f.^/f^ mice (Suppl. Figure 2e). Moreover, the increase in TfR1 protein was measured in HCC tissues in comparison to NTL (Fig. 3g-h), further corroborating the findings on dysregulated iron metabolism in p53-deficient liver carcinoma. In conclusion, CCl_4_-induced liver carcinogenesis in p53^LKO^ mutant mice results in accelerated hepatocarcinogenesis with tumors characterised by an iron-poor status and "high Tfr1 and low hepcidin" signatures.

**Fig. 4:**
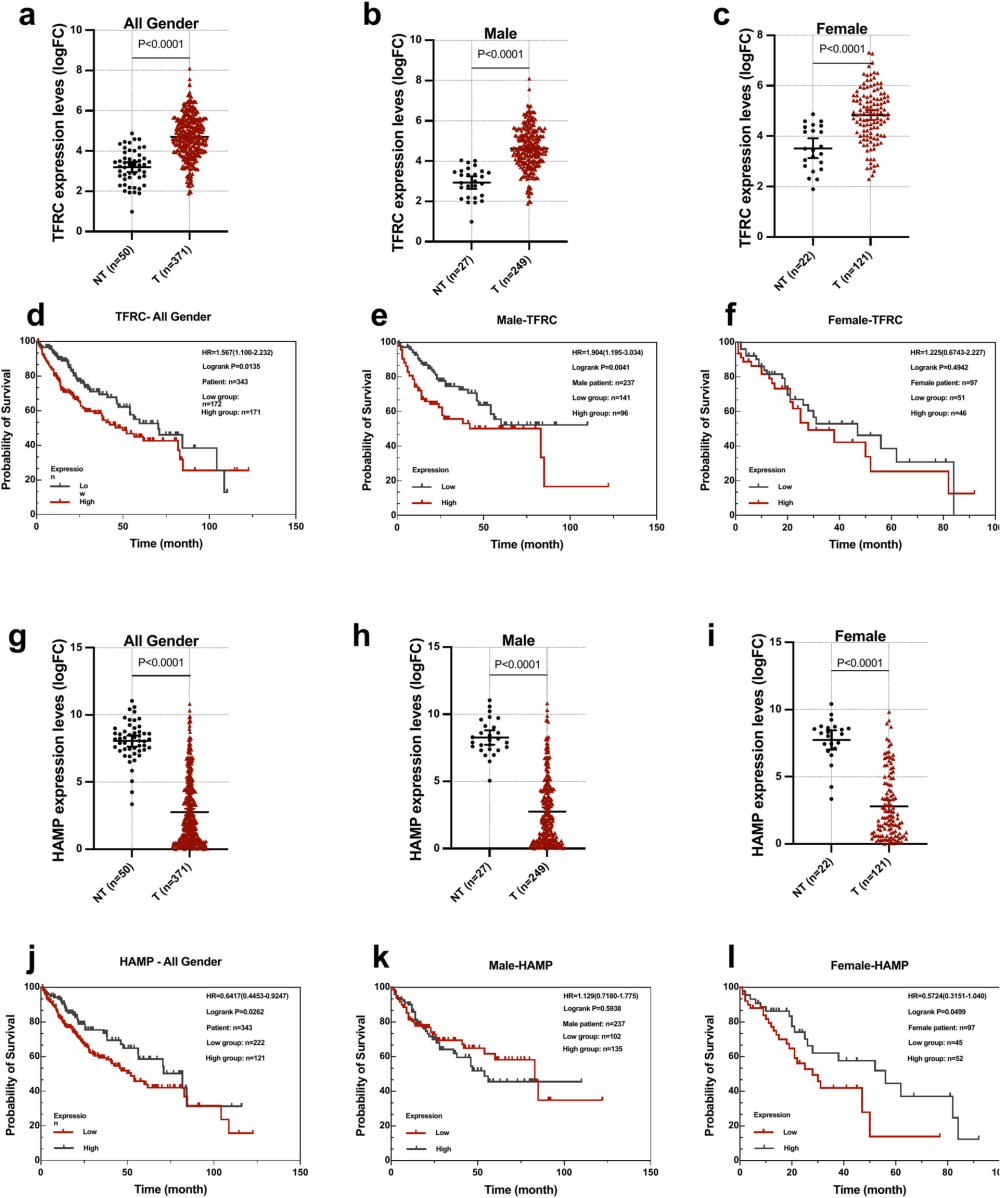
High transferrin receptor 1 and low hepcidin expression in liver carcinoma is associated with a poor prognosis. (**a-c**) *TFRC* expression levels in non-tumor (NT) and HCC (T) of human patients with (**d-f**) plots comparing probability of survival of *TFRC*^high^ and *TFRC*^low^ expressing HCC, in both genders, in male, and female patients, respectively. (**g-i**) *HAMP* expression levels in NT and T of human patients with (**j-l**) plot comparing probability of survival of *HAMP*^high^ and *HAMP*^low^ expressing HCC , in both genders, in male, and female patients, respectively.

We further accessed RNA-seq data from tumor tissues of 371 patients and 50 adjacent NTL available at The Cancer Genome Atlas (TCGA) database and inspected for changes in *TFR1* and *HAMP* mRNA levels. As illustrated in Fig. 4a-d, elevated *TFR1* and reduced *HAMP* mRNA levels were observed in tumor tissues in comparison to NTL, respectively. The same effect was observed when samples were stratified according to the patients’ sex (Fig. 4b-c, e–f). The overall survival curves demonstrated a significant correlation with high *TFR1* and low *HAMP* levels, indicating a poor prognosis for patients with these levels (Fig. 4a, d, top right panels). However, higher TFR1 expression significantly correlated with poor survival of male patients (Fig. 4b), while lower HAMP expression associated with worse prognosis in female patients (Fig. 4f). In conclusion, our data establish an iron-poor HCC phenotype in mice and patients which is characterised by "high Tfr1 and low hepcidin" iron signatures.

### Liver carcinogenesis correlates with systemic iron deficiency in p53^LKO^ female mice

To investigate whether iron-poor phenotype of liver cancer is a local phenomenon, confined to liver tumors, or whether systemic iron changes might be present in mice and potentially associate with liver carcinogenesis, we measured systemic iron parameters in CCl_4_-treated p53^LKO^ mice at 36 weeks of age and compared them to the iron status of p53f.^/f^ and healthy wild type mice of the same age.

**Fig. 5:**
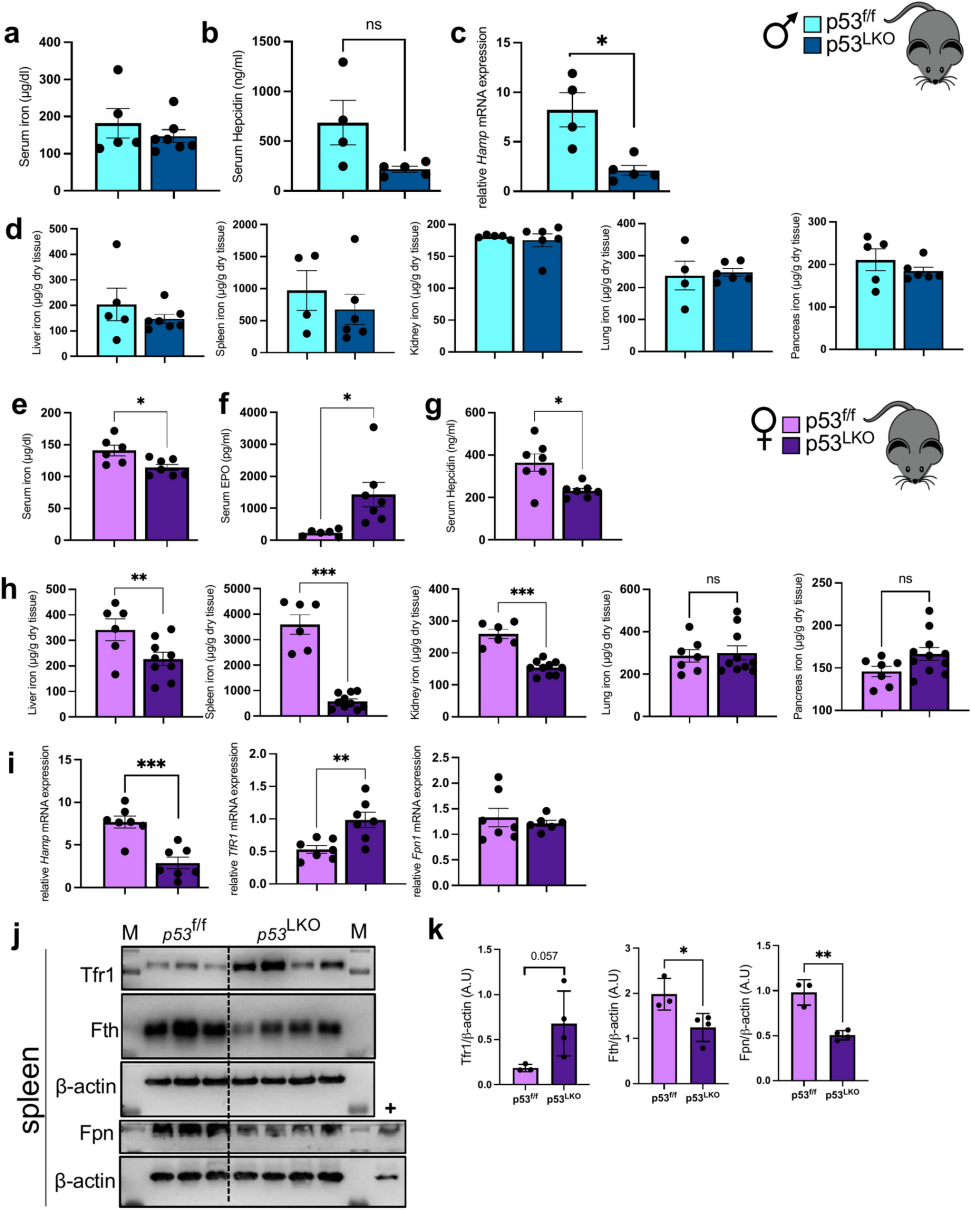
Hepatocellular carcinoma is characterized by a profound systemic iron deficiency in p53^LKO^ female mice. (**a**) Serum iron and (**b**) hepcidin levels in p53f.^/f^ and p53^LKO^ male mice. (**c**) Relative *Hamp* mRNA expression level in p53f.^/f^ and p53^LKO^ male mice. (**d**) Systemic iron levels in the liver , spleen, kidney, lung and pancreas of p53f.^/f^ and p53^LKO^ male mice. (**e-g**) Serum iron, erythropoietin (EPO) and hepcidin levels in p53f.^/f^ and p53^LKO^ female mice. (**h**) Systemic iron levels in the liver, spleen, kidney, lung, and pancreas of p53f.^/f^ and p53^LKO^ female mice. (**i**) Relative *Hamp, TfR1* and *Fpn1* mRNA expression level in the livers of p53f.^/f^ and p53^LKO^ female mice. (**j,k**) Representative immunoblot analysis and quantification blots of TFR1, FtH and Fpn relative to β-actin levels (expressed in arbitrary units) in the spleen of p53f.^/f^ and p53^LKO^ female mice. M: Molecular weight marker (kDa), + : positive control for Fpn expression

Here we demonstrate that male p53^LKO^ mice exhibit no significant changes in systemic iron parameters when compared to p53f.^/f^ and healthy wild-type mice (Fig. 5a-d; Suppl. Figure 2f., g). Surprisingly, and in contrast to male mice, liver carcinogenesis in female p53^LKO^ mice produces an overall systemic iron deficiency, characterized by low serum iron levels, high erythropoietin (EPO) levels, and low serum hepcidin that was accompanied by a decline in hepatic hepcidin mRNA expression, increase in TfR1 and no change in Fpn mRNA levels (Fig. 5e-i). Importantly, low iron content was detected in the liver, spleen and kidney (Fig. 5l), while lung and pancreas were not affected (Fig. 5m). Iron deficiency in the spleen was characterised by an increase in TfR1, and decrease in FtH and Fpn protein levels (Fig. 5j, k).

These results demonstrate that the loss of p53 not only affects tumor development and the expression of an iron-poor liver cancer phenotype, but greatly impacts systemic iron metabolism, resulting in the development of severe systemic iron deficiency in female p53^LKO^ mice.

## Discussion

Liver carcinoma is characterised by a spectrum of mutations affecting several pathways^38^. Whole-genome and exome sequencing of 243 human liver tumors identified the p53 gene as one of the most frequently mutated tumor-suppressor genes in hepatocellular carcinoma (HCC). Furthermore, over 50% of all cancer types are known to be associated with point mutations in the p53 gene^4–6^. In mice, liver-specific deletion of p53 induced tumors with high penetrance in 14–20-months old mice, demonstrating that p53 deletion, as a single genetic lesion in the liver, is sufficient to trigger tumor formation^10^. Here we show that CCl_4_-induced chronic liver damage in p53^LKO^ mice greatly enhanced liver cancer development as all mutant mice developed liver cancer by 36 weeks of age.

We further show that all liver carcinoma in p53^LKO^ mouse model exhibited low iron levels, low hepcidin expression, and high *Tfr1* expression. This led us to conclude that liver cancers display local iron perturbations and a state of iron deficiency, findings that align with previous studies in mice, rats and patients^29,30,32,39–43^. One potential explanation for the “high Tfr1”-signature in liver tumors is the ‘iron addiction’ of cancer cells, reflecting an increasing demand for iron by cancer cells, which is coupled with a high iron utilization/turnover. In contrast, the “low hepcidin” signature marks an iron-low state within tumors. In addition, low hepcidin may be related to poor tumor cell differentiation due to loss of p53^44^. In conclusion, these results show that liver tumor formation recruit pathways that enhance iron availability and the progressive utilisation of iron for subsequent cell division and tumor progression, without iron deposition in the HCC. Based on this data, we propose that a potential targeted strategy to ameliorate cancer incidence and progression, might be to interfere with transferrin-bound iron uptake by targeting the Tfr1 expression in hepatocytes. This idea is supported by three lines of observations demonstrating that *1)* silencing of *TFR1* in HepG2 cell lines, inhibited carcinogenesis^45^, that *2)* hepatic-*Tfr1* deficiency in mice reduced the uptake of transferrin-bound iron^46^, and that *3)* high *TFR1* expression in patients with primary liver tumors was associated with poor prognosis and survival (Fig. 4)^26^.

Our findings further indicate that iron deficiency along with selective deficits in other essential micronutrients is closely related to the progression of HCC, supporting previous meta-studies reporting lower levels of Fe and Zn in HCC than in the surrounding tissues^47^, while others have not demonstrated this effect^48,49^. We show the presence of low-Fe/low-Se levels in all liver cancers, while the distribution of Zn and Mn was different between males and females, suggesting that sex-specific differences are being relevant not only for the predisposition to the liver cancer development but also for the distribution of trace elements, like Zn and Mn. The underlying reason is currently unknown but given the presence of systemic iron deficiency in female mice, in addition to low iron within HCC, we speculate that, among other factors, the hormonal status, in particular the levels of steroid hormones such as estrogen and testosterone^50^, might contribute to the observed differences. Moreover, it is yet to be resolved whether low concentrations of trace elements are reduced because of impaired cellular uptake of trace elements, or due to the malignant transformation itself. Further studies are warranted to examine whether normalizing the cellular levels of trace elements, by supplementing dietary antioxidants and micronutrients, may improve the prognosis of liver cancer.

We believe that our experimental mouse model of CCl_4_-induced liver carcinogenesis in mice, which shows development of highly aggressive liver cancers with an iron-poor phenotype along with systemic iron dysregulation in female mice, represents an appropriate model system for future translational research. A limitation in our study is that we induced a complete loss of p53, which however, covers a broad spectrum of liver tumors with dysfunctional p53 signalling, loss of p53 function due to p53 mutations or by phenocopying p53 loss^51^. Conversely, the spectrum of p53 mutations occurring in liver cancer is not fully represented. Moreover, the loss of p53 is associated with undifferentiated liver tumors^44^ as well as the formation of metastasis^37^. However, when we look into liver cancer, the p53 cell cycle signalling pathway is altered in at least 50% of patients with hepatocellular carcinoma (HCC), a disease often associated with p53 mutations, occurring in 12% to 48% of cases^6^.

Lastly, our data might further be relevant for patients with HFE-based genetic hemochromatosis. Findings from the UK Bio Bank data demonstrated significantly increased risk for primary hepatic malignancy in 1294 male *HFE*-patients, compared to men with no pathogenic variants (7.2% vs. 0.6%)^52^. Two earlier studies in *HFE*-patients showed that *p53* was mutated in 71% of British *HFE* patients^53^ and that increased *p53* mutational load was present in livers of hemochromatosis patients^54^. Moreover, iron-free foci were observed in the livers of HFE-patients representing preneoplastic regions^34^, suggesting that iron-deficiency in liver cancers arising in HFE-background may possibly be due to effects mediated by the loss of p53, as demonstrated in our study. A combined deficiency of p53 and *HFE* in mice, may provide answers to liver cancer development and the regulation of iron homeostasis locally within HCC and at systemic levels. The model holds further potential to reveal whether the excess of iron, the lack of p53 in *HFE*-background, or both, are detrimental for the increased cancer risk in patients.

## Methods

### Ethics statement

Animal experiments were approved and performed in accordance with the University Animal Care Committee and the Federal Authorities for Animal Research (35/9185.81–3/1259, Regierungspraesidium Tuebingen, Baden-Wuerttemberg, Germany). All authors complied with the guidelines for animal research: reporting of in vivo experiments (ARRIVE).

### Mice

Mice with liver-specific Trp53 deficiency (abbreviated further as p53^LKO^) were generated as previously described^10^. As control mice we used Trp53flox/flox mice (short: p53f.^/f^) and healthy wild-type mice. Both, male and female mice were used in the study. All mice were maintained on a C57/BL6J background. At the age of six weeks, mice were injected intraperitoneally with 0.5 ml/kg bodyweight CCl_4_ (T5648-5G, Sigma-Aldrich) dissolved in olive oil (1:3) twice a week over a time period of 16 weeks to induce liver cirrhosis and subsequent carcinoma formation. The mice were euthanised by CO_2_ and subsequent cervical dislocation. The mice were euthanized in the home cage with subsequent neck dislocation. CO2 is gradually introduced into the home cage in accordance with Annex IV of Directive 2010/63/EU. The gas is introduced into the cage (type II long) via a flow meter through a tube. The flow rate is 20%, which corresponds to 1.7 L of CO_2_/min for a long type II cage. The weight of male p53f.^/f^ and p53^LKO^ mice at the time of euthanasia was 34.3 g + /- 2.9 g and 24.3 g + /- 2.1 g, respectively, and 27.3 g + /- 1.7 g and 23.7 g + /- 1.4 g in case of female p53f.^/f^ and p53^LKO^ mice, respectively. All mice were euthanised before a tumor reached the size of 15 mm in diameter (Fig. 1i).

### Serum parameters

Serum iron was determined using iron kit (Thermo Fisher Scientific, Finland) in a 96-well format using a serial dilution of iron atomic absorption standard solution (1000 mg/ml iron in HCl, Sigma-Aldrich, Inc.) according to manufacturer’s instruction. Iron values are expressed as µg of iron per deciliter (dl).

ALT (alanine aminotransferase) and AST (aspartate aminotransferase) were measured by using a Reflotron system (Roche Diagnostics, Mannheim, Germany).

### Liver and tumor histology

The liver and liver tumors were fixed in 4% paraformaldehyde (PFA) overnight at 4 °C, dehydrated in ethanol series and xylene, and embedded in paraffin. Sections were stained for H&E and for Picro-Sirius-Red and the positive red area was quantified with the ImageJ software (v.1.47); a color deconvolution plugin was used.

### Immunohistochemistry

Immunohistochemistry was performed on 2-µm-thick paraffin sections. Hepatocyte specific antibody, Hep Par1 (clone OCH1E5, Dako), was used for the detection of hepatocellular differentiation, and CK7 (Abcam, ab181598) for the detection of biliary differentiation. Antigen retrieval and immunostaining were carried out on a Dako-Omnis platform.

### Tissue iron measurement

The non-heme iron content in the liver and liver tumor tissues, as well in other organs was measured as previously described^55^.

### Total reflection X-ray fluorescence spectrometry

Total iron, manganese, zinc and selenium content in liver and liver cancer tissues was measured via total-reflection X-ray spectrometry (TXRF) as previously described^56^. Concentrations of trace elements are expressed in mg per mg P or μg per g dry tissue.

### Enzyme-linked immunosorbent assay (ELISA)

Hepcidin and Erythropoietin (EPO) levels were measured in mouse sera using HAMP ELISA kit (Intrinsic Lifesciences, USA) and EPO ELISA kit (R&D), according to the manufacturers’ instructions.

### Microarray analysis

Total RNA was isolated from whole liver carcinoma tissue. Samples with a RIN (RNA integrity number) value higher than 8.0 measured with a bioanalyzer (Agilent Technologies, Santa Clara, CA, USA) were used for further processing. Gene expression analysis was carried out using the SurePrint G3 Mouse Gene Expression 8 × 60 K Microarrays (Design ID 028,005; Agilent Technologies, Santa Clara, CA, USA). The samples were labelled with the Low Input Quick Amp Labelling Kit (Agilent Technologies, Santa Clara, CA, USA) according to the manufacturer’s guidelines. Slides were scanned using a G2565CA microarray scanner (Agilent Technologies, Santa Clara, CA, USA). Raw data files were extracted using the Feature Extraction software (v.10.7.3.1, Agilent Technologies, Santa Clara, CA, USA) and analysed with the Genespring software (Agilent Technologies, Santa Clara, CA, USA). Normalized intensity values and adjusted p-values were used for further plotting and analysis. For Gene-set enrichment analysis, GSEA-software (v. 4.3.2) was used. All gene expression data were deposited in Gene Expression Omnibus (GEO accession number GSE255470).

### RNA isolation, reverse-transcription and real-time PCR

Total RNA was isolated from snap frozen liver and tumor tissue using Trizol reagent (Life technologies, CA, USA) according to manufacturer`s instruction.

Reverse transcription and subsequent real-time PCR analysis were performed as previously described^55^. Primers used in the study are:

Gene Symbol Forward Sequence (5’-3’) Reverse Sequence (5’-3’).

Gapdh CCCATTCTCGGCCTTGACTGT GTGGAGATTGTTGCCATCAACGA.

Hamp ATACCAATGCAGAAGAGAAGG AACAGATACCACACTGGGAA.

Tfr1 CCCATGACGTTGAATTGAACCT GTAGTCTCCACGAGCGGAATA.

Smad7 GCAGGCTGTCCAGATGCTGT GATCCCCAGGCTCCAGAAGA.

Bmp6 GTGACACCGCCACAAC TCGTAAGGGCTCTCTG.

Hfe CACCGTCTGTGCCATCTTCTT ACATAGCCACCCATGGTTCCT.

### Protein isolation and Western blot analysis

Protein extracts were prepared from liver, HCC/ICC and spleen as previously described^55^. Membranes were blotted with mouse anti-transferrin receptor 1 (Tfr1; Zymed laboratories, USA; 1:500), rabbit-anti-Ferroportin (Alpha Diagnostics Ltd., USA; 1:500), and rabbit anti-ferritin-H (Cell Signaling Technology, USA; 1:1000 in 2% BSA) following manufacturer’s instructions. Membranes were washed and incubated with anti-rabbit or anti-mouse horseradish peroxidase-conjugated antibody (Invitrogen, USA; 1:5000). Reactions were carried out with Luminata Forte Western HRP substrate kit (Millipore, USA). As loading control anti-β-actin (Sigma Aldrich, USA; 1:10,000) was used. Membranes were washed prior to the addition of substrate and visualized in chemiluminescence detector (BioRad, USA).

### Bioinformatics analysis

RNA gene expression data and clinical information for liver cancer patients were obtained from the TCGA-LIHC project, comprising 371 liver cancer tissues and 50 adjacent non-tumor tissues from the GDC database (https://portal.gdc.cancer.gov/projects/TCGA-LIHC). Expression levels of TFRC and HAMP were analyzed using this dataset. The 367 patients with complete clinical data were categorized into high and low expression groups based on optimal cutoff values identified using the ‘*survminer’* R package. Kaplan–Meier survival curves and log-rank tests were then used to evaluate the relationship between TFRC and HAMP expression and overall survival (OS).

### Statistical analyses

Data were analysed using GraphPad Prism software and results are shown as mean ± standard deviation. For the statistical analysis, a non-parametric distribution and the Mann–Whitney-U test or two-tailed Student t test (with Welch´s correction) were used. Statistically significant differences are indicated as p ˂0.05 (*), p ˂0.01 (**) and p ˂0.005 (***).

## Supplementary Information

Supplementary Information.

## Data Availability

The data that support the findings of this study are available from the corresponding authors upon reasonable request. All gene expression data were deposited in Gene Expression Omnibus. The data can be accessed via the following webpage: https://www.ncbi.nlm.nih.gov/geo/query/acc.cgi?acc=GSE255470.
