# Supplementary material for: Iron metabolism in a mouse model of hepatocellular carcinoma
Source: Sci Rep. 2025 Jan 16;15:2180. doi: 10.1038/s41598-025-86486-x (PMC11739418; doi:10.1038/s41598-025-86486-x)
Supplement: Supplementary file 1 — Supplementary Information. [file 41598_2025_86486_MOESM1_ESM.docx]

**Supplementary Data**

***Iron metabolism in a mouse model of hepatocellular carcinoma***

Dilay Yilmaz, Umesh Tharehalli, Rossana Paganoni, Paul Knoop, Andreas Gruber, Yuexin Chen, Rui Dong, Frank Leithäuser , Thomas Seufferlein, Kerstin Leopold, André Lechel, and Maja Vujić Spasić

**Table of contents:**

| **Description** | **Title** | **Page number** |
| --- | --- | --- |
| **Supplementary Figure 1** | **Liver carcinoma induced by CCl_4_ in p53^LKO^ and p53^f/f^ mice** | **2** |
| **Supplementary Figure 2** | **Liver carcinoma formation in p53^f/f^ male mice results in Se and Zn deficiency without systemic iron changes** | **3** |


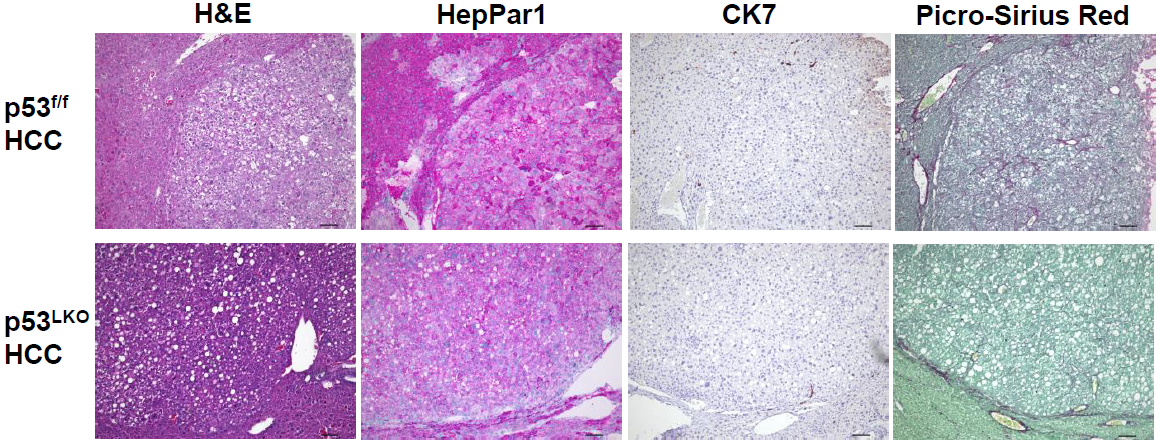


**Supplementary Figure 1. Liver carcinoma induced by CCl_4_ in p53^LKO^ and p53^f/f^ mice.**

Representative photographs of Hematoxylin/Eosin (H&E) staining, immunohistochemical staining for HepPar1 and CK7 (cytokeration 7), and Picro-Sirius-Red staining of hepatocellular carcinoma (HCC) from p53^LKO^ (upper row) and p53^f/f^ mice (bottom row).


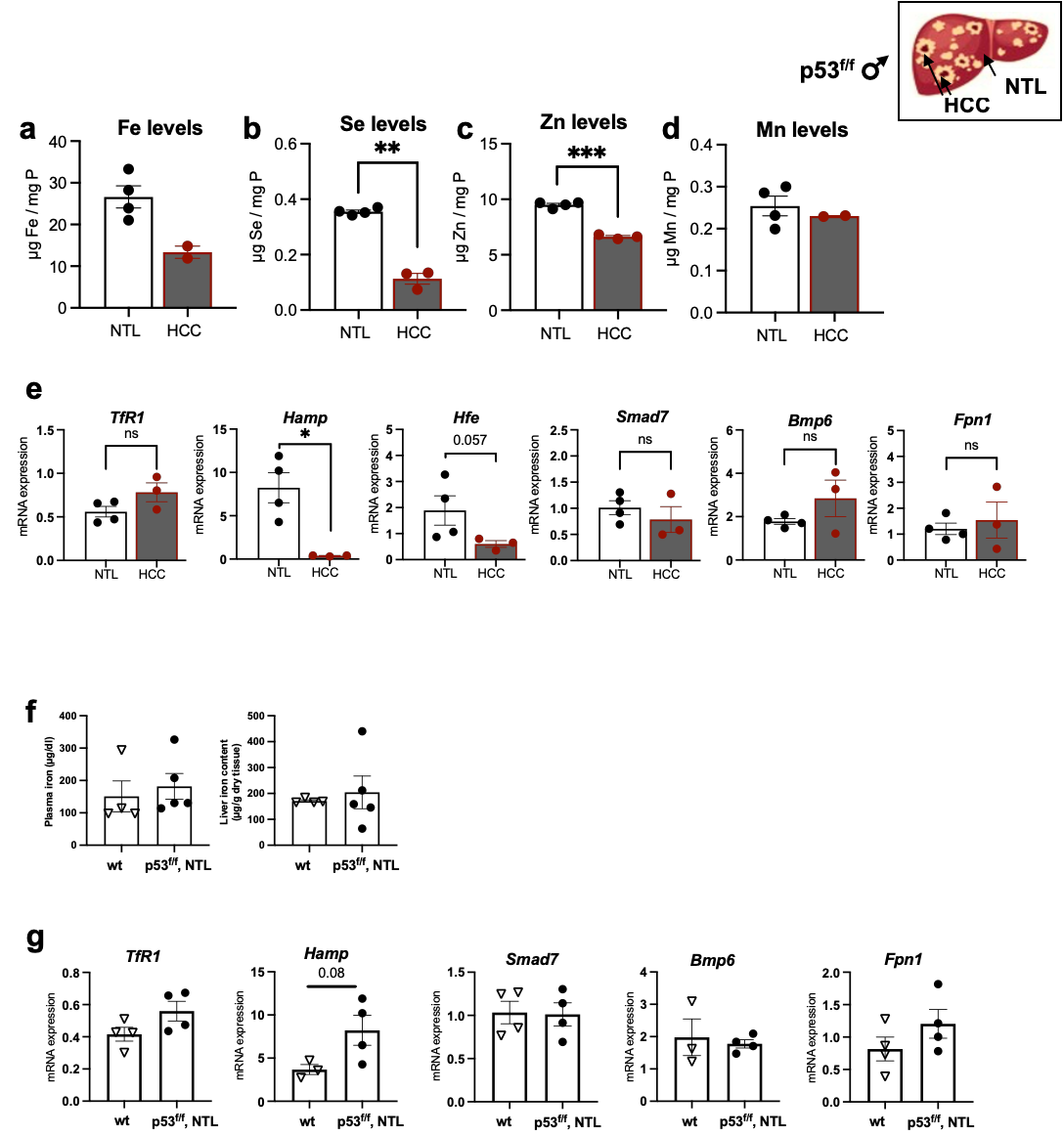


**Supplementary Figure 2. Liver carcinoma formation in p53^f/f^ male mice results in Se and Zn deficiency without systemic iron changes.**

(**a-d**) Total trace element content (iron, selenium, zinc and manganese) in NTL and HCC of p53^f/f^ male mice. (**e**) mRNA expression levels of *Tfr1*, *Hamp* (hepcidin), *Hfe*, *Smad7*, *Bmp6*, and *Fpn1* in NTL and HCC of p53^f/f^ male mice measured by qPCR. (**f**) Plasma and liver iron levels in healthy wild-type and p53^f/f^ male mice. (**g**) mRNA expression level of *Tfr1*, *Hamp*, *Smad7*, *Bmp6*, and *Fpn1* in the livers of healthy wild-type and p53^f/f^ male mice.
